# Evidence for a fragile X messenger ribonucleoprotein 1 (*FMR1*) mRNA gain‐of‐function toxicity mechanism contributing to the pathogenesis of fragile X‐associated premature ovarian insufficiency

**DOI:** 10.1096/fj.202200468RR

**Published:** 2022-10-17

**Authors:** Roseanne Rosario, Hazel L. Stewart, Nila Roy Choudhury, Gracjan Michlewski, Nicholas Charlet‐Berguerand, Richard A. Anderson

**Affiliations:** ^1^ MRC Centre for Reproductive Health, Queen's Medical Research Institute University of Edinburgh Edinburgh UK; ^2^ Biomedical Sciences University of Edinburgh Edinburgh UK; ^3^ Infection Medicine University of Edinburgh Edinburgh UK; ^4^ Zhejiang University‐University of Edinburgh Institute Zhejiang University Zhejiang P.R. China; ^5^ Dioscuri Centre for RNA‐Protein Interactions in Human Health and Disease International Institute of Molecular and Cell Biology in Warsaw Warsaw Poland; ^6^ Institut de Génétique et de Biologie Moléculaire et Cellulaire (IGBMC) INSERM U 1258, CNRS UMR 7104, Université of Strasbourg Illkirch France

**Keywords:** CGG trinucleotide repeats, FMRpolyG, FXPOI, mRNA gain‐of‐function

## Abstract

Fragile X‐associated premature ovarian insufficiency (FXPOI) is among a family of disorders caused by expansion of a CGG trinucleotide repeat sequence located in the 5′ untranslated region (UTR) of the fragile X messenger ribonucleoprotein 1 (*FMR1*) gene on the X chromosome. Women with FXPOI have a depleted ovarian follicle population, resulting in amenorrhea, hypoestrogenism, and loss of fertility before the age of 40. FXPOI is caused by expansions of the CGG sequence to lengths between 55 and 200 repeats, known as a *FMRI* premutation, however the mechanism by which the premutation drives disease pathogenesis remains unclear. Two main hypotheses exist, which describe an mRNA toxic gain‐of‐function mechanism or a protein‐based mechanism, where repeat‐associated non‐AUG (RAN) translation results in the production of an abnormal protein, called FMRpolyG. Here, we have developed an in vitro granulosa cell model of the *FMR1* premutation by ectopically expressing CGG‐repeat RNA and FMRpolyG protein. We show that expanded CGG‐repeat RNA accumulated in intranuclear RNA structures, and these aggregates were able to cause significant granulosa cell death independent of FMRpolyG expression. Using an innovative RNA pulldown, mass spectrometry‐based approach we have identified proteins that are specifically sequestered by CGG RNA aggregates in granulosa cells in vitro, and thus may be deregulated as consequence of this interaction. Furthermore, we have demonstrated reduced expression of three proteins identified via our RNA pulldown (FUS, PA2G4 and TRA2β) in ovarian follicles in a *FMR1* premutation mouse model. Collectively, these data provide evidence for the contribution of an mRNA gain‐of‐function mechanism to FXPOI disease biology.

**Abbreviations**
FMR1: Fragile X messenger ribonuceloprotein 1 gene
FXPOI: Fragile X‐associated premature ovarian insufficiency
FXTAS: Fragile X‐associated tremor and ataxia syndrome
RP‐SMS: RNA pulldown with high‐throughput mass spectrometry
SILAC: Stable isotope labelling by amino acids in cell culture

## 1 INTRODUCTION

The fragile X messenger ribonucleoprotein 1 (*FMR1*) gene is located on the X chromosome and contains a CGG trinucleotide repeat sequence within its 5′ untranslated region (5′UTR), expansions of which can result in both neurological and reproductive disorders. The polymorphic length of the repeat sequence is categorized into four different size ranges. Individuals who carry less than 44 CGG repeats have a normal repeat length that is usually transmitted in a stable manner from mother to offspring,^1^ while having between 45 and 54 repeats is classified as intermediate, or gray zone. Although gray zone repeat lengths are not directly associated with any disease phenotypes, some CGG repeat instability has been reported, which results in variable repeat expansion during transmission.^2^ Expansion of this CGG repeat sequence to more than 200 repeats is categorized as a full mutation, and underlies the severe neurodevelopmental condition fragile X syndrome,^3^ which is the most common cause of inherited intellectual disability and autism in males, with patients suffering from a wide range of clinical, cognitive and behavioral dysfunctions. The range of 55–200 CGG repeats is considered a premutation, and some females with this develop what is now known as fragile X‐associated premature ovarian insufficiency (FXPOI).^4^, ^5^ Premutations also result in the more recently described fragile X‐associated tremor/ataxia syndrome (FXTAS), a multisystem neurological disorder with tremor and ataxia as its principal features, which was initially recognized in aging carriers but with clinical features potentially also present in children.^6^

Premature ovarian insufficiency (POI) is defined by the depletion of the ovarian follicle population, resulting in amenorrhea, hypoestrogenism, and loss of fertility before the age of 40 years.^7^ In addition to the direct impact on fertility, secondary consequences arising from estrogen deficiency compromise bone, cardiovascular and neurological health of affected individuals (comprehensively reviewed in Ref. [8]). Despite advances in genomic technologies and the strides taken to unravel the genetic determinants of POI, abnormalities in the *FMR1* gene are the only monogenic cause currently tested for in routine clinical practice. Approximately 20–30% of female premutation carriers develop FXPOI,^9^, ^10^ with these women having midrange CGG tract sizes between 70 and 100 repeats.^9^, ^11^ However, an additional ~20% of females with the premutation present with irregular periods and a further ~13% report difficulty conceiving.^12^, ^13^ Even premutation carriers without signs of ovarian dysfunction have a menopause that is on average 5 years earlier than women in the general population,^11^ thus although the premutation does not necessarily result in POI, it is clear that its presence impairs normal *FMR1* gene function in the ovary with a range of clinical consequences.

The mechanisms that underlie compromised ovarian follicular function preceding the full development of FXPOI are unclear, but it is proposed these insults could occur at various stages of follicular development. In the normal ovary, *FMR1* is thought to regulate ovarian follicle recruitment,^14^ however the ovarian mRNA targets of the FMRP RNA binding protein are unknown. Findings from knock‐in mouse models^15^, ^16^, ^17^ generally show consensus in their reproductive physiology and demonstrate that the *FMR1* premutation allele does not interfere with the establishment of the primordial follicle pool. However, the population of growing follicles exhibited increased atresia, affecting all growing follicle stages.^15^, ^16^ This follicle decline was paralleled by a decrease in litter size.^18^ Although how this results in premature depletion of the ovarian reserve (i.e., non‐growing follicle pool) is unclear, there are clear interactions between the growing and non‐growing pools that regulate the activation of follicle growth.^19^, ^20^

**FIGURE 1:**
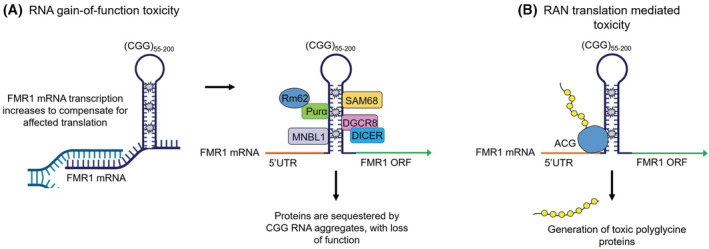
Proposed models of *FMR1* premutation toxicity. (A) RNA gain‐of‐function toxicity. *FMR1* transcription increases to compensate for affected translation. Subsequently, premutation CGG repeat lengths form intranuclear aggregates that can sequester RNA binding proteins, inhibiting them from carrying out their normal roles, leading to cell dysfunction. (B) Repeat‐associated non‐AUG (RAN) translation mediated toxicity. Translation of *FMR1* mRNA is initiated from a near cognate ACG start codon, resulting in the production of polyglycine and/or polyalanine‐containing proteins that interfere with normal cell function or might directly be toxic. Figure taken from Ref. [21].

At a molecular level, FXPOI shares many common features with the other premutation associated disorder, FXTAS, and advances made in understanding this neurological condition have also been applied to the pathogenesis of FXPOI (reviewed in Ref. [21]). In premutation carriers, the *FMR1* locus is transcriptionally active and mRNA levels are elevated.^22^ Thus, a key hypothesis is that *FMR1* mRNA gain‐of‐function toxicity may underlie FXPOI, a concept that originated from the pathogenesis of another trinucleotide expansion disease myotonic dystrophy (see Figure 1A).^23^, ^24^ In this model, *FMR1* transcription is augmented and expanded CGG‐containing transcripts accumulate into nuclear RNA foci, which bind and sequestrate specific RNA‐binding proteins and thus potentially inhibit their normal functions, compromising cell functions.^25^, ^26^, ^27^, ^28^, ^29^, ^30^, ^31^, ^32^, ^33^ It is important to note that in this model, toxicity arises because of the expanded CGG repeat itself, and not of overexpression of *FMR1* protein product, as overexpression of *FMR1* mRNA without a CGG repeat expansion does not trigger neuronal death or produce behavioral deficits.^34^ A second (and non‐exclusive) model has been proposed recently, based on the observation that expanded repeat sequences can be translated in absence of any AUG canonical start codon, through a mechanism named repeat‐associated non‐AUG (RAN) translation (see Figure 1B).^35^, ^36^ In the case of *FMR1*, expanded CGG repeats are predominantly translated into a polyglycine‐containing protein, named FMRpolyG, which forms ubiquitin‐positive intranuclear inclusions and which expression is toxic for neurons in cell and animal models.^37^, ^38^, ^39^ Intranuclear inclusions of FMRpolyG have been detected in the brains of FXTAS patients,^37^, ^39^, ^40^, ^41^ as well as in non‐CNS tissues,^42^ including the ovarian stroma of a woman with FXPOI,^43^ and in mural granulosa cells from six *FMR1* premutation carriers.^44^ Furthermore, FMRpolyG protein has been detected in the ovarian stroma of mice expressing expanded CGG repeats.^43^, ^45^ Collectively these data suggest that RAN translation may be involved in FXPOI. Conversely, there is also evidence that an RNA gain of function toxicity mechanism contributes to FXPOI, as FMRP is expressed in granulosa cells of mature follicles in adult ovaries^46^ and increased *FMR1* transcript levels have been reported in granulosa cells of premutation carriers.^47^ Moreover, expression of CGG‐repeat RNA in mouse ovary leads to ovarian abnormalities.^15^, ^16^, ^45^ Thus, it is currently unclear whether FXPOI is caused by CGG RNA repeats, expression of FMRpolyG or a mix of both mechanisms.^15^

To study the relative contributions of mRNA gain‐of‐function toxicity and RAN translation in the pathogenesis of FXPOI, we established an in vitro human granulosa cell line model of the *FMR1* premutation by ectopically expressing CGG‐repeat RNA and FMRpolyG protein. This enables us to study the molecular basis of this disease, which may have late‐onset effects causing ovarian follicle loss in premutation carriers precluding the study of endogenous CGG‐repeats and FMRpolyG protein. Given the limitations of this overexpression system, we have used a FXPOI mouse model to confirm findings in an endogenous CGG repeat model. We found that expanded CGG‐repeat RNA accumulated in intranuclear structures, and using an innovative methodology that combines RNA pulldown with stable isotope labelling by amino acids in cell culture (SILAC) high‐throughput mass spectrometry (RP‐SMS), we identified proteins that are specifically sequestered by CGG RNA aggregates in granulosa cells in vitro. We have shown colocalisation of three of these endogenous proteins with CGG RNA aggregates (FUS, PA2G4 and TRA2β) and demonstrated reduced expression of these proteins in ovarian follicles from FXPOI mice. Lastly, CGG‐repeat RNA caused significant levels of granulosa cell death, which was independent of the presence of FMRpolyG protein. These data thus provide evidence for the contribution of the mRNA gain‐of‐function mechanism to FXPOI disease, and provide protein targets whose dysregulation may contribute to this pathological condition.

## 2 METHODS

### 2.1 Plasmids

Plasmids expressing 60 CGG repeats (referred to as 60x CGG) or 100 CGG repeats within the human *FMR1* sequence, fused to GFP without and with the ACG start codon deleted (referred to as Δ5′UTR *FMR1* (CGG)100x GFP and 5′UTR *FMR1* (CGG)100x GFP, respectively) have been described previously.^30^, ^39^ For flow cytometry‐based cell viability assays, a plasmid was created by inserting GFP with a CMV promoter and terminator sequence (amplified from pEGFP) into the Δ5′UTR *FMR1* (CGG)100x GFP plasmid^39^ using BglII and EcoRI restriction sites (referred to as Δ5′UTR *FMR1* (CGG)100x GFP_GFP). All CGG plasmids were transformed into NEB® Stable Competent *E. coli* (New England Biolabs, UK) and grown at 30°C according to manufacturer's instructions.

### 2.2 Cell culture and transient transfections

HGrC1^48^ and COV434^49^ cells were cultured in DMEM/F‐12 (Gibco™, ThermoFisher Scientific, UK) supplemented with 10% fetal bovine serum (Gibco™) and maintained at 37°C in 5% CO_2_. For transient transfections to express 60x CGG, 5′UTR *FMR1* (CGG)100x GFP or Δ5′UTR *FMR1* (CGG)100x GFP plasmids or to overexpress GFP‐ or HA‐tagged proteins of interest, cells were seeded at density of 80 000 cells per well of a 4‐well chamber slide (Nunc, ThermoFisher Scientific). Single and double transfection experiments were carried out using Lipofectamine 3000 (Invitrogen, ThermoFisher Scientific) according to manufacturer's instructions.

### 2.3 RNA fluorescence in situ hybridisation (FISH) combined with immunocytochemistry

Chamber slides with transfected cells were fixed in 4% paraformaldehyde in PBS (pH 7.4) for 15 min at room temperature. Cells were permeablised with 0.5% Triton X‐100/PBS for 5 min, and washed in PBS before pre‐hybridisation in 40% DMSO (Sigma‐Aldrich, UK), 40% formamide (Sigma‐Aldrich), 10% BSA (10 mg/ml) and 2x saline‐sodium citrate (SSC) for 30 min in a humidified hybridisation oven set to 60°C. Chamber slides were hybridized for 2 h in 40% formamide, 10% DMSO, 2x SSC, 2 mM vanadyl ribonucleotide (Sigma‐Aldrich), 60 mg/ml yeast RNA (ThermoFisher Scientific), 30 mg/ml BSA plus 0.75 μg (CCG)_8x_‐Cy3 DNA oligonucleotide probe (Integrated DNA Technologies, UK). Following hybridisation, the chamber slides were washed twice successively at 55°C in 2x SSC/50% formamide and 2x SSC, and counterstained with 4,6‐diamidino‐2‐phenylidole (DAPI) before mounting in Permafluor Aqueous Mounting Medium (Perkin‐Elmer, ThermoFisher Scientific). To confirm the RNA composition of CGG aggregates, treatment with RNAase A (Roche Diagnostics, UK) was carried out according to manufacturer's instructions prior to permeablisation. If immunocytochemistry was carried out immediately following in situ hybridisation, instead of counterstaining, chamber slides were washed three times in PBS before incubation with primary antibody (diluted in PBS) overnight at 4°C. Primary antibody dilutions were: anti‐GFP at 1:400 (ab6556, Abcam), anti‐HA at 1:500 (clone 16B12, #MMS‐101P, Covance), anti‐FUS at 1:400 (AMAb90549, Atlas Antibodies), anti‐PA2G4 at 1:50 (15348‐1‐AP, Proteintech), anti‐TRA2β at 1:800 (ab31353, Abcam) and anti‐SQSTM1 (p62) at 1:500 (ab91526, Abcam). Immunocytochemistry was also carried out for expression of FUS, PA2G4 and TRA2β independently of CGG FISH, and for colocalisation of FMRpolyG and p62, antibodies were co‐incubated overnight at 4°C. The next day, chamber slides were washed in PBS before incubation with an Alexa Fluor 488‐conjugated secondary antibody at 1:200 (Molecular Probes) for 60 min at room temperature. Chamber slides were then counterstained with DAPI before mounting. Slides were imaged using either a Zeiss LSM 780 confocal microscope (Carl Zeiss, Oberkochen, Germany) or an Axioscan slide scanner (Carl Zeiss). For colocalisation analyses, Z‐stack images were acquired with a Zeiss LSM 780 confocal microscope using the correct Nyquist sample rate and deconvolved using Huygens Essential. The 3D ImageJ Suite in FIJI was used to carry out segmentation and calculate percentage colocalisation using the middle slice of the Z stack.

### 2.4 Immunohistochemistry

Immunohistochemistry for FUS, PA2G4,TRA2β, MSY2 and AMH was carried out on PFA‐fixed paraffin‐embedded ovarian sections from 6‐month‐old wildtype and CAG LoxP 5′UTR *FMR1* (CGG)99x GFP x CMV Cre bigenic mice,^39^ according to standard protocols. Primary antibody incubations were carried out overnight at 4°C with dilutions as follows: anti‐FUS at 1:100 (AMAb90549, Atlas Antibodies), anti‐PA2G4 at 1:100 (15348‐1‐AP, Proteintech), anti‐TRA2β at 1:400 (ab31353, Abcam), anti‐MSY2 at 1:1000 (Ab82527, Abcam) and anti‐AMH at 1:100 (sc‐166 752, Santa Cruz). Secondary antibody labelling and detection was carried out at room temperature as follows: for FUS and AMH—Mouse on Mouse Polymer IHC Kit (ab269452, Abcam) and Opal Fluorophore Reagent (Akoya Biosciences, MA, USA) according to manufacturer's instructions, for PA2G4 and MSY2—anti‐rabbit peroxidase at 1:200 for 30 min (PI‐1000, Vector Labs) followed by Opal Fluorophore Reagent (Akoya Biosciences), and for TRA2β an Alexa Fluor 488‐conjugated secondary antibody at 1:200 (Molecular Probes) was used for 60 min. Tissue was counterstained with DAPI before mounting. Slides were imaged using an Axioscan slide scanner (Carl Zeiss), with quantification of staining intensity carried out in FIJI. Mean gray values of FUS, PA2G4 or TRA2β were normalized to the mean gray value of MSY2 or AMH for oocyte and granulosa cell data, respectively.

### 2.5 RNA pulldown SILAC high‐throughput mass spectrometry (RP‐SMS) and Western blotting

RNA pulldown coupled to stable isotope labelling by amino acids in cell culture (SILAC) mass spectrometry was carried out as described previously.^50^ Briefly, HGrC1 cells were cultured in SILAC media (DC Biosciences, Dundee, UK), ‘heavy’ or ‘light’, supplemented with dialysed calf serum (DC Biosciences) to incorporate cells with heavy or light isotopes. Cell extracts were prepared and incubated with CGG_(30x)_ RNA (Dharmacon, Cambridge, UK) coupled to agarose beads. Following a series of washes to remove unbound protein, proteins were electrophoresed into an SDS‐PAGE gel (Bio‐Rad, Watford, UK), and submitted for LC–MS/MS analysis performed using an Orbitrap™ mass spectrometer (ThermoFisher Scientific). Data was analyzed using the MaxQuant software^51^ to determine the ratio of heavy‐labeled peptides to light‐labeled peptides, and identify proteins specifically bound to CGG RNA. Pulldown experiments followed by Western blotting were used to validate mass spectrometry data. Pulldown was carried out as described above with CGG_(30x)_ RNA, pre‐let‐7a‐1 RNA (generated via in vitro transcription^50^) or beads only, and proteins were separated on an SDS‐PAGE gel (Bio‐Rad). Proteins were transferred onto Immobilon FL membrane (Millipore, Dorset, UK), which was blocked using Intercept blocking buffer (LI‐COR Biosciences, Cambridge, UK). Western blotting was undertaken with anti‐FUS (AMAb90549), anti‐PA2G4 (15348‐1‐AP) and anti‐TRA2β (ab31353) antibodies, at a dilution of 1:1000, 1:250 and 1:500, respectively, overnight at 4°C. Alexa Fluor 680‐ and 800‐conjugated secondary antibodies (Molecular Probes) were used for detection (at 1:10 000) and blots were imaged on a LI‐COR FC Odyessy. Western blotting was also undertaken to confirm that the 5′UTR *FMR1* (CGG)100x GFP plasmid was translated into FMRpolyG protein using a mouse monoclonal antibody specific to FMRpolyG N‐terminal sequence (MEAPLPGGVRQRGGG, antibody clone 8FM^39^) and to assess alterations in FUS, PA2G4 and TRA2β expression following transfection of an empty, Δ5′UTR *FMR1* (CGG)100x GFP or 5′UTR *FMR1* (CGG)100x GFP plasmids into HGrC1 cells using the same protocol. Quantification of band intensity was carried out using Image Studio v5.2, with normalization of FUS, PA2G4 and TRA2β expression to ACTB (A2066, Sigma‐Aldrich, used at 1:1000) or TUBA (T6074, Sigma‐Aldrich, used at 1:1000) as loading controls.

### 2.6 MTT cell viability assay

HGrC1 cells (maintained in phenol‐free DMEM/F12 + 10% FBS) were seeded at a density of 20 000 cells per well of a 96 well plate and transfected with either an empty plasmid, (CGG)60x, Δ5′UTR *FMR1* (CGG)100x GFP or 5′UTR *FMR1* (CGG)100x GFP as described above. At 72 h post transfection, 10 μl of 12 mM of MTT (thiazolyl blue tetrazolium bromide, M5655, Sigma) was added to each well, and the plate incubated at 37°C for four hours. A permeabilisation solution (10% SDS in 0.01 M HCl) was then added to each well, with the plate incubated for a further four hours. Absorbance at 570 nm was measured using a Labtech LT‐4500 plate reader.

### 2.7 Flow cytometry cell viability assay

HGrC1 and COV434 cells were seeded at a density of 300 000 cells per well of a 6 well plate and transfected with either an empty pEGFP plasmid, Δ5′UTR *FMR1* (CGG)100x GFP_GFP or 5′UTR *FMR1* (CGG)100x GFP as described above. At 72 h post transfection, media was collected for floating cells and this was combined with cells that were trypsinised and neutralized, with 6 wells pooled per condition in order to have enough cells for flow cytometry analysis. Just before analysis, cells were incubated with DAPI (1:1000) for 3 min as a cell viability marker. Flow cytometry was carried out on a BD LSRFortessa™ and data analyzed using BD FACSDiva™ software (version 8.0). Single cells were analyzed for GFP expression (to identify positively transfected cells) and DAPI staining, with DAPI positive cells indicative of a compromised cell membrane, thus poor cell viability.

### 2.8 RNA extraction, cDNA synthesis and RT‐qPCR

To assess the expression of granulosa cell genes in HGrC1, COV434 and Ishikawa (ISHI) cell lines, RNA was extracted using the RNeasy mini kit (Qiagen) according to manufacturer's instructions. RNA was reverse transcribed to cDNA using concentrated random primers and Superscript III reverse transcriptase (ThermoFisher Scientific) according to manufacturer's instructions, and the cDNA synthesis reaction was diluted appropriately before proceeding. Primers for quantitative RT‐PCR (RT‐qPCR) were designed to amplify all transcript variants and are exon‐spanning. Each reaction was performed in a final volume of 10 μl, with 1× Brilliant III SYBR Green qPCR Master Mix (Agilent, UK), 20 pmol of each primer and 2 μl of diluted cDNA. Primer sequences are as follows written in the 5′ to 3′ direction: *FOXL2* F TACTCGTACGTGGCGCTCAT, *FOXL2* R CTCGTTGAGGCTGAGGTTGT, *FSHR* F GCTGCCTACTCTGGAAAAGC, *FSHR* R ATCTCTGACCCCTAGCCTGA, *CYP19A1* F TCACTGGCCTTTTTCTCTTGGT, *CYP19A1* R GGGTCCAATTCCCATGCA, *RPL32* F CATCTCCTTCTCGGCATCA, *RPL32* R AACCCTGTTGTCAATGCCTC, *RPLP0* F ATGGGCAAGAACACCATGATG and *RPLP0* R CCTCCTTGGTGAACACAAAGC. Each cDNA sample was analyzed in triplicate. Target genes were normalized to the geometric mean expression of *RPL32 and RPLP0*. Data analysis for relative quantification of gene expression and calculation of standard deviations was performed as outlined.^52^, ^53^

### 2.9 Statistical analysis

All data are shown as mean ± standard error of the mean and were analyzed using GraphPad Prism 9 software (GraphPad Software, Inc., San Diego, CA). Mann–Whitney and Freidman test statistics were carried out as appropriate. A *p* value of <.05 was considered statistically significant.

## 3 RESULTS

### 3.1 Expanded CGG repeats within the *FMR1* 5′UTR form intranuclear RNA aggregates and FMRpolyG protein aggregates in granulosa cell lines

**FIGURE 2:**
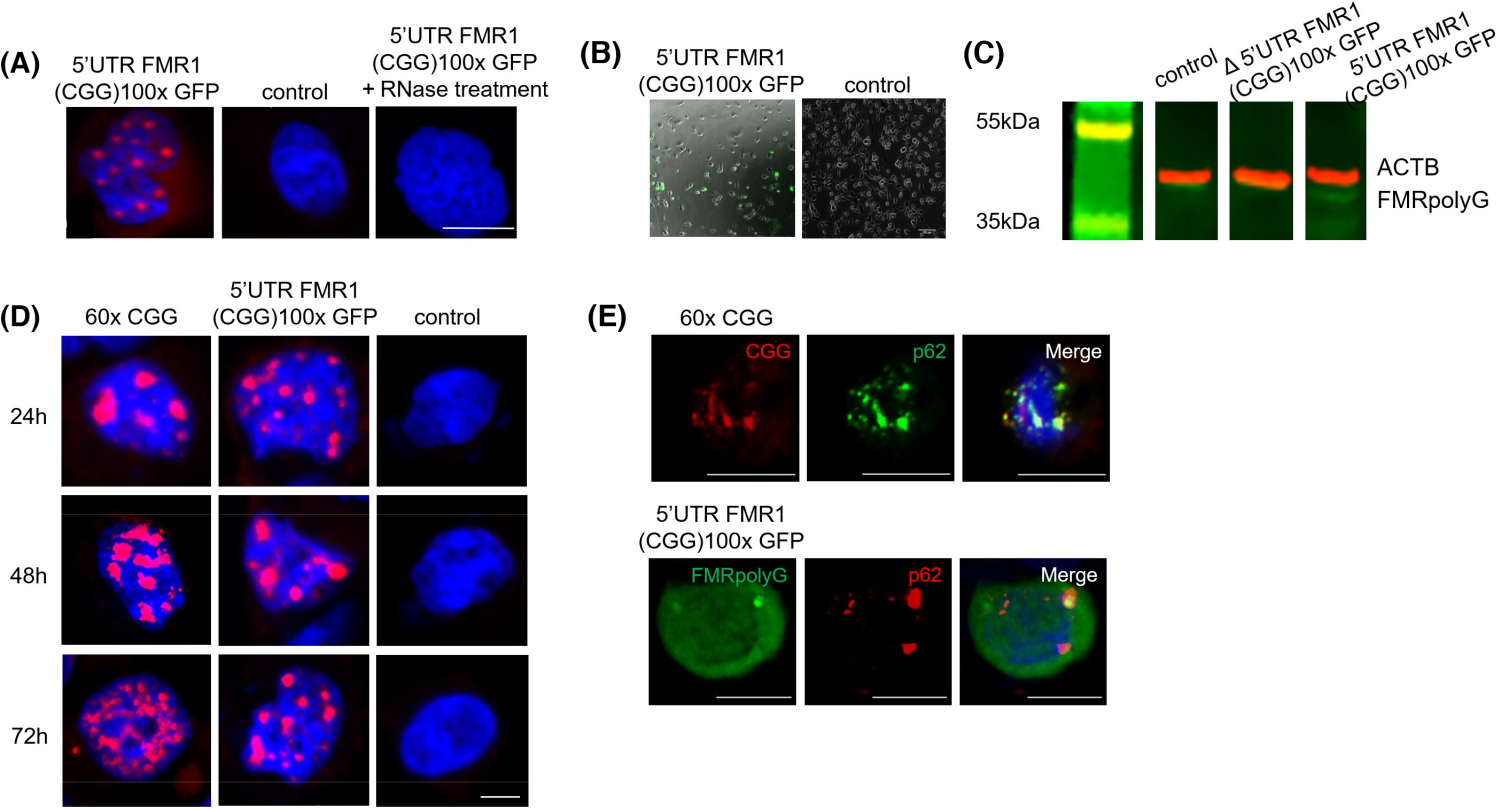
Expanded CGG repeats within the *FMR1* 5′UTR form intranuclear RNA aggregates and FMRpolyG protein aggregates in granulosa cell lines. HGrC1 cells were transfected with a plasmid expressing either 100 CGG repeats within the *FMR1* 5′UTR or no CGG repeats (control) and analyzed 24 h after transfection by RNA FISH using a (CCG)8x‐Cy3 DNA probe counterstained with DAPI or fluorescence microscopy for the presence of CGG RNA aggregates (A) or FMRpolyG protein (B), respectively. (C) Western blotting using an FMRpolyG‐specific antibody^39^ following transfection of Δ5′UTR FMR1 (CGG)100x GFP and 5′UTR FMR1 (CGG)100x GFP plasmids confirms only the latter is capable of producing FMRpolyG protein, with a band (green) visible at ~37–40 kDA, corresponding to FMRpolyG itself and a GFP tag. ACTB (red) was used as a loading control. (D) HGrC1 cells were transfected with a plasmid expressing either 60x CGG repeats, 100 CGG repeats within the *FMR1* 5′UTR or no CGG repeats (control) and analyzed at 24, 48 and 72 h after transfection by RNA FISH. Whilst RNA aggregates formed following expression of 60x CGG repeats increased in size and number over time, RNA aggregates formed following expression of the 5′UTR FMR1 (CGG)100x GFP plasmid were stable in size and number. (E) Immunostaining for p62 expression in CGG‐RNA aggregate‐positive and FMRpolyG‐positive cells. Scale bars represent 10 μM.

To investigate the consequences of the *FMR1* premutation in granulosa cells, we transfected a plasmid expressing 100 CGG repeats embedded within the 5′UTR of the human *FMR1* gene and fused to the GFP in the glycine frame into two granulosa cell lines HGrC1 and COV434, and tested the formation of CGG RNA foci and FMRpolyG expression using RNA fluorescence in situ hybridisation (FISH) and fluorescence microscopy, respectively. Expression of this plasmid generated numerous intranuclear CGG RNA foci in both granulosa cell lines, which could be observed at 24 h post transfection (Figures 2A and S1A). The RNA composition of these aggregates was confirmed as they were sensitive to RNase A treatment. Both granulosa cell lines were also able to translate this plasmid into FMRpolyG protein, as GFP‐tagged protein was observed at 24 h post transfection (Figures 2B and S1B). Western blotting was used to confirm that the observed GFP‐tagged protein was indeed FMRpolyG as a band ~37‐40 kDa was observed in 5′UTR *FMR1* (CGG)100x GFP transfected cells, corresponding to FMRpolyG itself (~12‐14 kDa with 100 CGG repeats) fused to the GFP (~25 kDA) (Figure 2C). This band was not observed in cells transfected with an empty plasmid or the Δ5′UTR *FMR1* (CGG)100x GFP plasmid, which is unable to produce FMRpolyG due to deletion of its ACG near‐cognate start codon.^39^

As CGG RNA foci are observed in some cell lines (e.g., COS7), but not in other (e.g., HEK293, HeLa, A172, U‐937 etc., see^30^), we confirmed these results using a second plasmid, which expresses 60 CGG repeats in isolation under the control of a CMV promoter. These repeats are deleted of their natural *FMR1* sequence and thus cannot express the FMRpolyG protein, where initiation occurs at near‐cognate codons located upstream of the repeats within the 5′UTR of *FMR1*. RNA foci dynamics were analyzed by RNA FISH at 24, 48 and 72 h after transfection. HGrC1 cells were transfected with either the 5′UTR *FMR1* (CGG)100x GFP plasmid or the (CGG)60x construct. The expressed 60 CGG repeats formed intranuclear RNA aggregates that increased in size and number over time (Figure 2D), as reported in COS7 cells.^30^ In contrast, the intranuclear CGG RNA aggregates that formed as a result of the expression of the 5′UTR *FMR1* (CGG)100x GFP plasmid were more stable and did not evolve in size or number over time (Figure 2D). Similar findings were observed when these experiments were carried out in COV434 cells (Figure S1C). Next, we carried out immunostaining for p62, a marker of the proteasomal and autophagic degradation pathways and observed that some, but not all cells, positive for either CGG RNA or FMRpolyG expression were also positive for p62 at 48 h post transfection (Figure 2E). This suggests that the CGG RNA aggregates and FMRpolyG expression observed are not artefactual but biologically meaningful.^40^, ^41^, ^54^ It is possible that the proportion of p62 positive CGG/FMRpolyG positive cells may increase at 72 h post transfection, however this was not explored further due to low cell viability at later time points.

**FIGURE 3:**
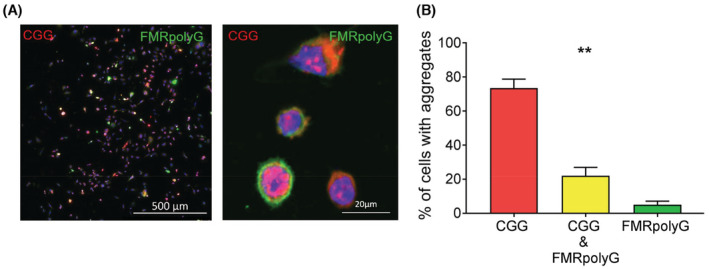
Expanded CGG‐repeat mRNA is not efficiently translated into FMRpolyG protein in HGrC1 cells. HGrC1 were transfected with a plasmid expressing 100 CGG repeats within the *FMR1* 5′UTR and RNA FISH followed by GFP immunocytochemistry were used 48 h after transfection to identify the colocalisation of CGG RNA aggregates and FMRpolyG protein expression. A representative image is shown in (A). Scale bars represent 500 and 20 μM, respectively. Quantification of cells expressing either CGG RNA only, CGG RNA and FMRpolyG or FMRpolyG only in HGrC1 (B) Data are presented as the mean ± SEM of four individual experiments. Friedman test, **p = .0046.

That CGG expanded repeats embedded in the natural *FMR1* sequence robustly formed RNA foci in HGrC1 cells was unexpected, given that it has been shown that this mRNA should be exported into the cytoplasm for translation into FMRpolyG protein.^39^ Therefore, we quantified CGG RNA foci and FMRpolyG expression in HGrC1 by transfecting cells with the 5′UTR *FMR1* (CGG)100x GFP plasmid and using RNA FISH followed by GFP immunocytochemistry to detect CGG RNA foci and FMRpolyG expression in the same cells at 48 h after transfection (representative image of this analysis is shown in Figure 3A). In HGrC1 cells, most transfected cells were positive for RNA foci only (73.5% ± 5.3%), with a smaller proportion (22.0% ± 2.5%; *p* = .0046) of cells expressing both CGG RNA foci and FMRpolyG protein and very few expressing FMRpolyG protein only (Figure 3B). Taken together, the expression of premutation length CGG repeats in granulosa cells and the variable translation of these repeats into FMRpolyG protein, is consistent with a CGG RNA gain‐of‐function model contributing to FXPOI pathogenesis.

### 3.2 CGG‐repeat RNA and FMRpolyG protein affect granulosa cell viability equally

**FIGURE 4:**
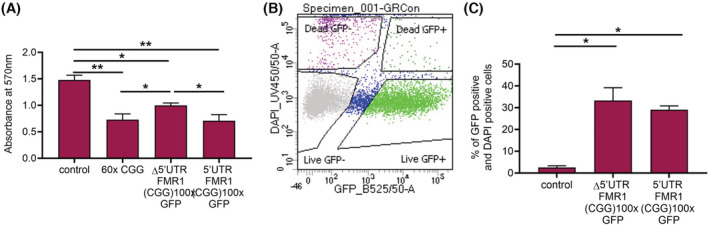
HGrC1 cell viability following expression of CGG‐repeat RNA only or CGG‐repeat RNA and FMRpolyG. (A) HGrC1 cells were transfected with an empty plasmid, (CGG)60x plasmid, Δ5′UTR *FMR1* (CGG)100x GFP plasmid, or 5′UTR *FMR1* (CGG)100x GFP plasmid, and an MTT assay was carried out at 72 h post transfection to assess cell viability. (B) HGrC1 cells were transfected with an empty pEGFP plasmid, Δ5′UTR *FMR1* (CGG)100x GFP_GFP or 5′UTR *FMR1* (CGG)100x GFP and collected for analysis via flow cytometry at 72 h post transfection. A representative image of gating for GFP and DAPI positive single cells in shown in (B). Quantification of GFP and DAPI positive HGrC1 cells (C). Data are presented as the mean ± SEM of four individual experiments, Mann–Whitney test, **p < .004 *p < .028.

It was apparent that transfection of CGG‐repeat RNA and FMRpolyG expressing plasmids affected cell viability, thus we carried out an MTT assay to explore the individual effects of CGG‐repeat RNA and FMRpolyG protein on the viability of HGrC1 cells (Figure 4A). To do this, we transfected cells with either an empty plasmid as a negative control, the (CGG)60x plasmid which produces CGG‐repeat RNA, the Δ5′UTR *FMR1* (CGG)100x GFP construct, which only produces CGG‐repeat RNA within the context of *FMR1*, or the 5′UTR *FMR1* (CGG)100x GFP plasmid, which is transcribed and translated into FMRpolyG‐GFP. At 72 h post transfection, all three plasmids caused at least a 30% decrease in viability compared to control transfected cells (*p* < .02) (Figure 4A). There was also a small but significant difference in cell viability between (CGG)60x and Δ5′UTR *FMR1* (CGG)100x GFP transfected cells (*p* = .028) and Δ5′UTR *FMR1* (CGG)100x GFP and 5′UTR *FMR1* (CGG)100x GFP transfected cells (*p* = .014). Given that this MTT assay was carried out on all cells within the well, these findings do not represent the viability of only positively‐transfected cells, and it is possible that differences in plasmid transfection efficiency were confounding the results.

Therefore to circumnavigate this, we developed a flow cytometry‐based cell viability assay using GFP to isolate positively transfected cells and DAPI as a marker of cell viability. An example of our gating strategy is depicted in Figure 4B. Given the artificial nature of the CGG(60x) plasmid, we omitted this plasmid from our analysis and transfected cells with either a construct expressing GFP alone as a negative control, the 5′UTR *FMR1* (CGG)100x GFP plasmid, which produces GFP‐tagged FMRpolyG, or the Δ5′UTR *FMR1* (CGG)100x GFP_GFP construct, which produces CGG‐repeat RNA and GFP separately to allow selection of the transfected cells by FACS. We carried out this experiment in HGrC1 and COV434 cells. At 72 h post transfection, both CGG‐repeat RNA and FMRpolyG protein caused significant cell death in both cell lines compared to cells expressing GFP only (*p* = .028) (Figures 4C and S2). However, there was no significant difference in the proportion of dead cells expressing CGG‐repeat RNA only vs. FMRpolyG (33.3% ± 5.9% vs. 29.1% ± 1.8% in HGrC1 cells, and 21.0% ± 0.9% vs. 30.4% ± 4.3% in COV434 cells). Given that CGG‐repeat RNA resulted in similar levels of cell death with or without FMRpolyG, this suggests that accumulation CGG‐repeat RNA alone can cause granulosa cell loss and play a part in FXPOI disease biology, supporting an mRNA gain‐of‐function toxicity model, while not excluding a contribution of FMRpolyG.

### 3.3 RNA pulldown‐SILAC mass spectrometry (RP‐SMS) identifies proteins that bind CGG aggregates

**FIGURE 5:**
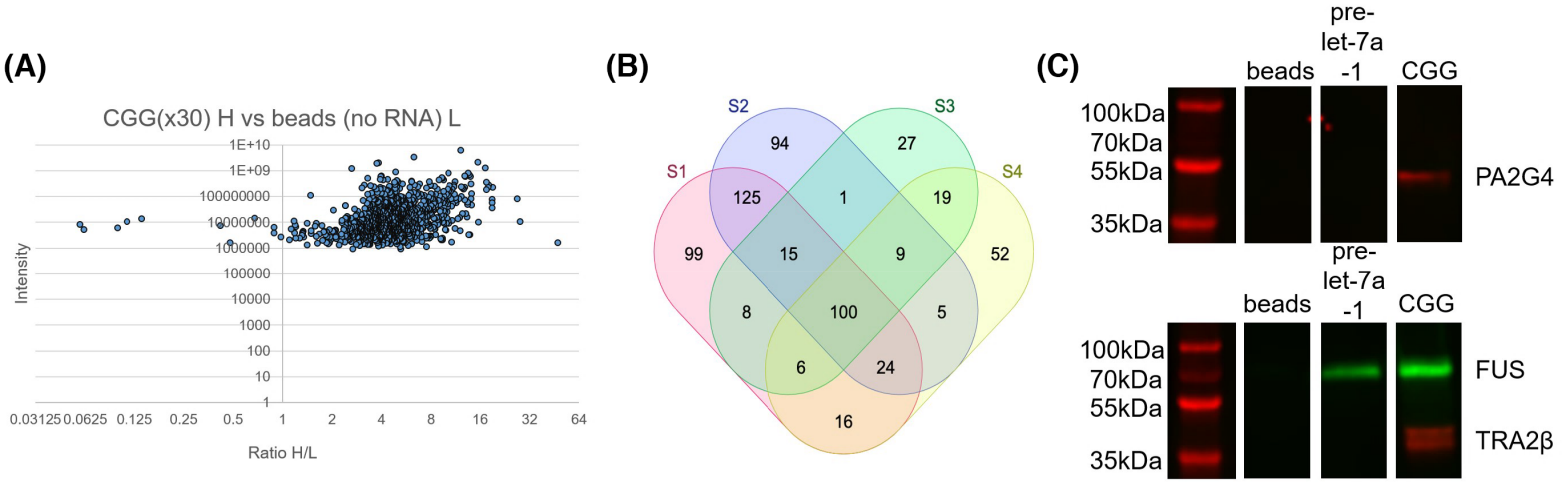
RP‐SMS identifies proteins that bind CGG RNA aggregates in HGrC1 cells. (A) A representative distribution of H/L ratios among proteins identified in the CGG_30x_ RNA pulldown. Results reveal that most proteins identified bind specifically to CGG_30x_ RNA as opposed to non‐specifically to beads, i.e., with 2‐fold or more enrichment. (B) Venn diagram depicting the overlap between proteins identified in four replicate RP‐SMS experiments, where two had ‘heavy’‐labeled RNA and two had ‘light’‐labeled RNA. 100 proteins are strong binders that are enriched 7‐fold or more in all four experiments. (C) Western blot investigating the specificity of FUS, PA2G4 and TRA2β binding to CGG_30x_ RNA, with beads‐only and pre‐let‐7a‐1 RNA as controls.

As CGG‐repeat RNA caused significant granulosa cell death, we used a novel methodology that combines RNA pulldown with SILAC high‐throughput mass spectrometry (RP‐SMS^50^) to identify proteins that are associated with CGG repeats, which could potentially be sequestered by RNA foci in granulosa cells and become dysregulated causing cell dysfunction and ultimately cell death. HGrC1 cells were grown in ‘light’ R0K0 medium or in ‘heavy’ R6K4 (13C labeled arginine and 2D labeled lysine) medium. Next, RNA pull‐down was performed with either agarose beads incubated with extract from light or heavy cells or beads with CGG30x RNA covalently linked incubated with extract from light or heavy cells, respectively. After thorough washing, the resulting supernatants were mixed and subjected to quantitative mass spectrometry. A representative distribution of heavy/light (H/L) ratios among proteins identified in the CGG30x RNA pulldown in shown in Figure 5A. Results reveal that most proteins identified bind specifically to CGG RNA as opposed to non‐specifically to the beads, i.e., were enriched more than 2‐fold (see File S1). 100 proteins were consistently identified in all four of the CGG30x RNA pulldown experiments as strong binders with an enrichment of 7 or more, as shown in the Venn diagram (Figure 5B). This figure depicts the overlap between proteins with an enrichment of 7‐fold or more compared to beads alone identified in four replicate RP‐SMS experiments, where two had ‘heavy’‐labeled proteins incubated with RNA and two had ‘light’‐labeled proteins incubated with RNA. Although we only used 30 CGG RNA repeats in our RP‐SMS experiment, which is not within the premutation range of repeats, it is widely acknowledged that increasing lengths of trinucleotide repeats do not bind different RNA binding proteins, but rather, more of the specific proteins that bind to shorter repeats.^55^, ^56^ Furthermore, this approach with only 8 CGG repeats has successfully identified proteins bound to CGG repeats that may be involved in FXTAS disease progression.^30^ Although the CGG‐repeat sequence is not very specific and this methodology was used as a screen to identify potential disease‐driving candidates, it was reassuring that among the proteins we identified were many known RNA splicing and RNA binding proteins, including hnRNPH proteins and MBNL1, that have previously been reported to associate with CGG RNA in mouse brain and COS7 cells.^30^ Among the proteins identified, two were of particular interest: FUS, which is an RNA binding protein involved in amyotrophic lateral sclerosis and DNA damage repair^57^ and PA2G4 (also named ErbB3‐binding protein 1 [EBP1]) that binds ribosomal RNA involved in cell proliferation.^58^ Disruption of these two cellular functions could lead to granulosa cell death and subsequent follicle loss as observed in FXPOI. As a positive control, we also tested TRA2β as this RNA splicing factor was shown to co‐localize in COS7 cells expressing premutation length CGG RNA and has been hypothesized to be involved in FXTAS disease progression.^30^, ^31^

RNA pulldown experiments were repeated with inclusion of pre‐let‐7a‐1 RNA followed by Western blotting to examine the specificity of binding of FUS, PA2G4 and TRA2β to CGG‐repeat RNA. Pre‐let‐7a‐1 was selected as an additional control due to its 72 nucleotide size similarity with CGG30x and analysis of previous RP‐SMS data^50^ identified an overlap of only 29 proteins enriched at least two‐fold (H/L ratio ≥ 2) between the pre‐let‐7a‐1 dataset and our CGG dataset (File S2). While binding of PA2G4 and TRA2β appeared to be specific to CGG RNA, with no bands observed in the beads‐only or pre‐let‐7a‐1 controls, FUS was found to bind both RNAs tested (Figure 5C), however this was not surprising as FUS was identified in the pre‐let‐7a‐1 RP‐SMS dataset.

### 3.4 FUS, PA2G4 and TRA2β cellular localisation and overall expression is affected by CGG‐repeat RNA expression

**FIGURE 6:**
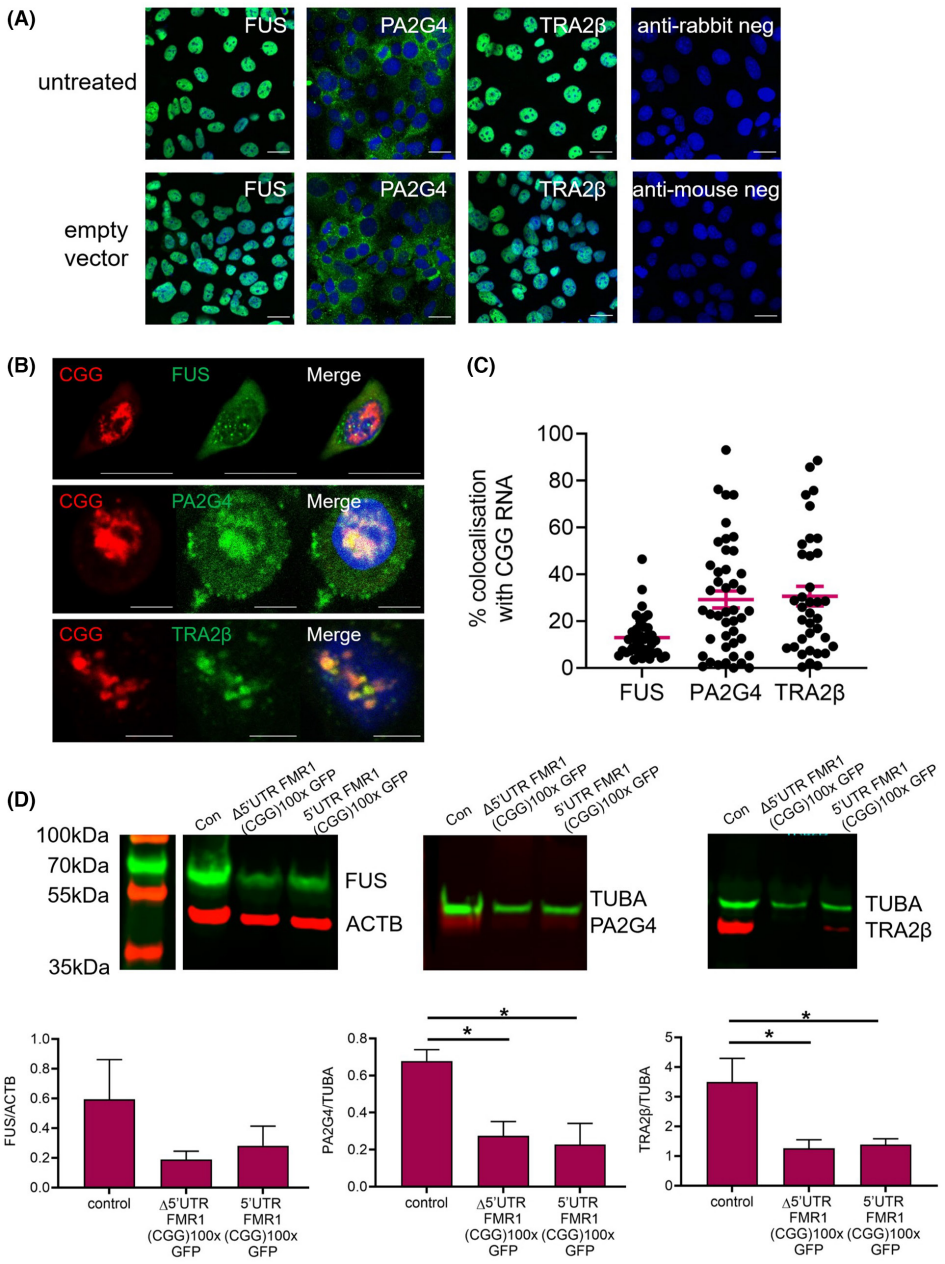
Quantification of co‐localisation and overall expression of endogenous FUS, PA2G4 and TRA2β in CGG‐repeat RNA and FMRpolyG transfected HGrC1 cells. (A) HGrC1 cells were transfected with an empty plasmid or left untreated, and immunocytochemistry was used at 48 h post transfection to examine the cellular localisation of candidate proteins. Scale bars represent 20 μM. (B) HGrC1 cells were co‐transfected with a plasmid expressing 60x CGG repeats and RNA FISH followed by immunocytochemistry were used after 48 h to identify the colocalisation of CGG RNA aggregates and candidate proteins. Scale bars represent 10 μM. (C) Quantification of colocalisation from 40 individual cells over three separate experiments. Data are presented as the mean ± SEM. (D) Western blot for candidate protein expression following transfection of empty, Δ5′UTR *FMR1* (CGG)100x GFP or 5′UTR *FMR1* (CGG)100x GFP plasmids. Quantification of signal intensity is normalized to that of loading control ACTB or TUBA. Data are presented as the mean ± SEM of four individual experiments, Mann–Whitney test, *p < .05.

Next, we tested for co‐localisation of these candidates with RNA aggregates in HGrC1 cells co‐transfected with 60 CGG repeats and GFP‐ or HA‐tagged proteins, combining FISH with immunocytochemistry at 48 h post transfection; the negative effects on cell viability (Figure 4) precluded these experiments from being undertaken 72 h post transfection. We observed that the presence of CGG RNA changed the intracellular distribution of FUS, PA2G4 and TRA2β expression inside the cell with some areas of co‐localisation (see Figure S3A). To further confirm the binding of FUS, PA2G4 and TRA2β with CGG RNA repeats, we quantified the co‐localisation of CGG RNA with endogenously expressed FUS, PA2G4 and TRA2β in HGrC1 cells using antibodies specific to each protein and compared the expression pattern to untransfected cells or those transfected with an empty plasmid (Figure 6A,B). In untransfected and control‐transfected cells, FUS and TRA2β expression was nuclear, while PA2G4 was observed in the cytoplasm; there was no significant change in the cellular localisation of these proteins between the two experimental groups (Figure 6A). However, upon expression of CGG‐repeat RNA, we consistently observed evidence of translocation of FUS and PA2G4 from their normal cellular location in the nucleus and cytoplasm, to the cytoplasm and nucleus, respectively, with areas of colocalisation observed between CGG‐repeat RNA and each of the three candidate proteins (Figure 6B). Results from a 2D analysis of 40 individual HGrC1 cells from over three repeated experiments, showed on average 13.0%, 29.3% and 30.7% co‐localisation of CGG‐repeat RNA with FUS, PA2G4 and TRA2β, respectively (Figure 6C). This co‐localisation tended to be highly variable, which may be a consequence of decreasing cell viability. Similar experiments were carried out in COV434 cells, which showed 21.5%, 8.7% and 45.5% co‐localisation of CGG‐repeat RNA with FUS, PA2G4 and TRA2β, respectively (Figure S3B,C).

Lastly, we examined whether there were any overall changes in FUS, PA2G4 and TRA2β expression in HGrC1 cells in the presence of CGG‐repeat RNA or FMRpolyG. Western blotting of FUS, PA2G4 or TRA2β endogenous expression was undertaken at 48 h post transfection of empty, Δ5′UTR *FMR1* (CGG)100x GFP or 5′UTR *FMR1* (CGG)100x GFP plasmids. Following normalization to loading controls, there was a significant decrease in PA2G4 and TRA2β expression in the presence of CGG‐repeat RNA and FMRpolyG (Figure 6D). Expression of PA2G4 was approximately 40% and 33% of that measured in control‐transfected cells (*p* < .05) while expression of TRA2β was approximately 36% and 40% of control‐cell levels (*p* = .028), with transfection of Δ5′UTR *FMR1* (CGG)100x GFP or 5′UTR *FMR1* (CGG)100x GFP plasmids, respectively. No significant changes were observed in FUS expression levels.

### 3.5 FUS, PA2G4 and TRA2β protein expression is reduced in ovarian follicles in a *FMR1* premutation mouse model

**FIGURE 7:**
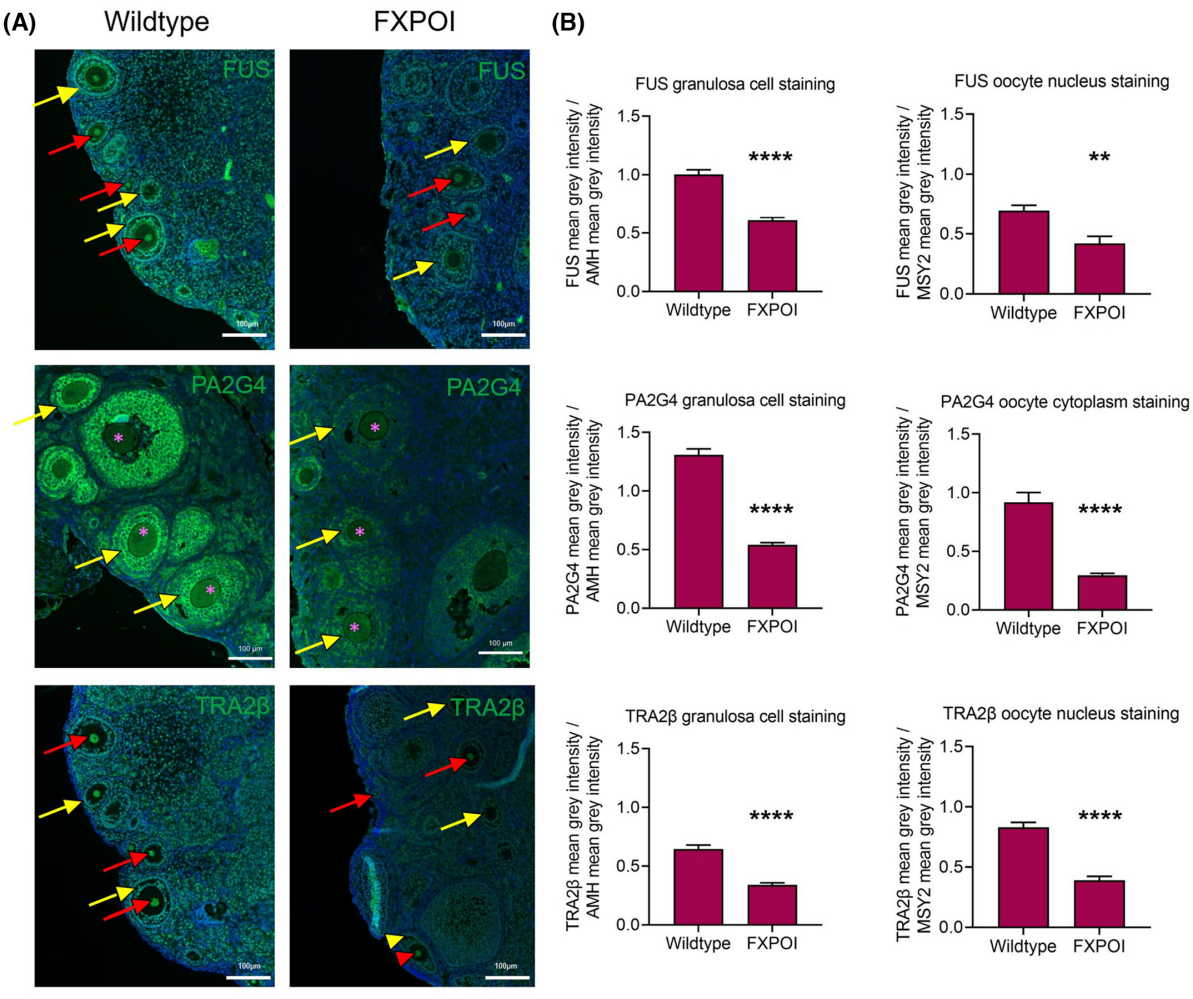
Expression of FUS, PA2G4 and TRA2β in 6 month old wildtype and FXPOI mice. (A) Representative images of FUS, PA2G4 and TRA2β expression in 6 month old wildtype and CAG LoxP 5′UTR *FMR1* (CGG)99x GFP x CMV Cre bigenic mice (referred to as FXPOI mice). Oocyte nuclei and cytoplasm are denoted with a red arrow head and pink asterisks, respectively, and granulosa cells, with a yellow arrow head. Scale bars represent 100 μM. (B) Quantification of mean gray values for FUS, PA2G4 and TRA2β representative of staining intensity were normalized to mean gray values of AMH (for granulosa cell data) and MSY2 (for oocyte data). Data are presented as the mean ± SEM from three separate mice. Mann–Whitney test, ****p < .0001 **p = .002.

Having identified these proteins using our RP‐SMS approach and observing their changes in cellular localisation and overall expression with CGG‐RNA repeats and FMRpolyG expression in a human granulosa cell line in vitro, we sought to investigate whether there were any differences in the expression of FUS, PA2G4 and TRA2β in the ovaries of a *FMR1* premutation mouse model. We analyzed ovaries from six‐month‐old CAG LoxP 5′UTR *FMR1* (CGG)99x GFP x CMV Cre bigenic mice, which ubiquitously express CGG‐repeat RNA and FMRpolyG,^39^ as well as age‐matched wildtype mice (Figure 7A). Quantification of FUS, PA2G4 and TRA2β staining intensity using mean gray values (MGV) was normalized to AMH and MYS2 staining intensities, given that these are characteristic granulosa cell and oocyte markers respectively (and not identified as CGG‐interacting proteins), and they did not show any significant differences between wildtype and FXPOI ovaries (Figure S4). The analysis showed that all three proteins were significantly less abundant in granulosa cells in premutation ovaries from three separate mice compared to wildtype controls, and there was no relationship to ovarian follicle stage (FUS: 40.3 vs. 26.0 MGV, PA2G4: 52.6 vs. 23.1 MGV, TRA2β: 25.9 vs. 14.4 MGV, *p* < .0001 for all proteins) (Figure 7B). Interestingly, we also observed a decrease in FUS (42.8 vs. 25.8 MGV), PA2G4 (56.4 vs. 18.2 MGV) and TRA2β (50.99 vs. 24.0 MGV) oocyte staining intensity compared to wildtype controls (*p* = .002 for FUS and *p* < .0001 for PA2G4 and TRA2β), which may suggest that oocytes in FXPOI ovaries are also compromised, which may have a role in ovarian follicle death.

## 4 DISCUSSION

Much of the research into the molecular mechanisms underlying the pathology of *FMR1* premutation‐associated conditions has focussed on the neurological aspects. This has led to two main hypotheses being proposed, which describe an RNA gain‐of‐function or a RAN translation‐based mechanism to explain how the *FMR1* premutation drives the pathogenesis of these disorders. In this study, we sought to investigate whether these hypotheses could explain the ovarian dysfunction observed in FXPOI. To do this, we expressed CGG‐repeat RNA, with and without accompanying FMRpolyG (the RAN translation protein product) in two human granulosa cell lines HGrC1 and COV434 and explored the consequences of this. The HGrC1 cell line is derived from granulosa cells of antral follicles,^48^ and to our knowledge, is the only human cell line that possesses characteristics of granulosa cells belonging to early stage follicles. Other granulosa cell lines have been derived from follicles after in vitro fertilization, and are therefore luteinised, or have been established from granulosa cell tumors. This was thought to be the case for COV434, although recent findings have questioned the true origin of this cell line,^59^ thus the present data using HGrC1 cells may be more relevant to ovarian follicular function (see Figure S5 for an RT‐qPCR characterization of HGrC1 and COV434 cells). Whilst we acknowledge this in vitro model does not truly recapitulate premutation granulosa cells found in vivo, the scarcity of FXPOI patient tissue with ovarian follicles available for study necessitates this compromise. Furthermore, this cell‐based model enables the study of human‐specific disease mechanisms where expanded CGG‐repeat expression is restricted to one cell type.

Following expression of 60x or CGG‐repeat RNA, deprived of its natural FMR1 5′UTR sequence, in these granulosa cell lines, we observed the formation of intranuclear CGG RNA foci that increased in size and number over a 72 h period. This finding has been described in other cell lines,^30^ however expression of microsatellite repeats in isolation, without their natural sequence in which they are normally embedded, can lead to a nuclear retention bias where the mRNA is not exported into the cytoplasm for translation and accumulates in the nucleus instead.^39^ Therefore, we additionally expressed 100 CGG repeats embedded in its natural *FMR1* 5′UTR sequence, which resulted in large stable RNA aggregates. This observation is interesting as we anticipated this mRNA to be exported and RAN translated into FMRpolyG protein. Indeed, CGG RNA aggregates are rare occurrences in brain tissue from transgenic mice engineered to express 99 CGG repeats within the human 5′UTR *FMRI* gene,^39^ and other knock‐in mouse models expressing premutation‐length CGG repeats.^30^ Given that our data show that HGrC1 cells had an accumulation of CGG RNA aggregates which then resulted in cell death, we suggest that an mRNA gain‐of‐function mechanism is pertinent to FXPOI pathogenesis.

In support of the mRNA gain‐of‐function hypothesis, numerous studies have made efforts to identify the various proteins that can be sequestered by CGG‐repeat mRNA and subsequently potentially deregulated or displaced from their physiological RNA targets.^26^, ^27^, ^28^, ^30^ Furthermore, the CGG‐repeat mRNA is known to adopt secondary structures such as intramolecular hairpins that may recruit specific RNA binding proteins.^60^ However, this work has mostly been undertaken in relation to FXTAS models of disease, and thus identified candidate proteins with relevance only to neuronal cells. Here we used a novel RNA pulldown method that enabled us to identify proteins that specifically bind to CGG‐repeat RNA in granulosa cells, making this the first study to investigate this RNA‐protein interaction in the ovary. Ideally, pulldown experiments to seek CGG RNA interactants should be undertaken using cellular material isolated from FXPOI patients, but given that premutation granulosa cells are usually only isolated after IVF treatment, it is difficult to ascertain how representative those cells are of early stage follicular cells. Instead, we chose to use HGrC1 granulosa cells that readily form CGG RNA foci upon transfection. Some of the candidate proteins identified here, such as TRA2β, MBNL1, and DDX5, were also identified in brain‐based studies.^26^, ^27^, ^30^ Of our candidate proteins, none have established connections to ovarian cell biology, thus we validated FUS and PA2G4 given their well‐characterized roles in DNA damage repair^57^ and cell proliferation^58^ respectively, and TRA2β given its hypothesized involvement in FXTAS disease progression.^30^ DNA damage repair is emerging as a key process in determining the ovarian lifespan, i.e., age at both premature and normal menopause^61^, ^62^ and overexpression of FUS in a drosophila model of FXTAS increased the toxicity phenotype of CGG‐repeat RNA.^33^ We hypothesized that sequestration of these proteins and subsequent deregulation of their function could be potentially detrimental for granulosa cells, and this could underlie the follicle loss that characterizes POI. Although highly variable, we observed similar levels of colocalisation of PA2G4 and TRA2β with CGG‐repeat RNA, with lower levels of co‐localisation for FUS. These lower levels may be explained by the fact that there was consistent evidence of FUS translocation to the cytoplasm in the presence of CGG‐repeat RNA, and indeed FUS has been shown to accumulate in the cytoplasm following DNA damage, leading to cellular apoptosis.^63^ Redistribution of FUS to the cytoplasm requires stress granule formation^64^ and the consequences of this for mRNA processing and translation in granulosa cells would be a worthwhile direction for future investigations. Given that FUS was also shown to bind pre‐let‐7a‐1 RNA and did not show overall changes in expression following CGG‐repeat RNA or FMRpolyG expression, unlike PA2G4 and TRA2β, it may be that the interaction between FUS and CGG‐repeat RNA is not completely specific, with involvement of other regulatory mechanisms. Given the toxicity of CGG repeats to these granulosa cell lines, it was not possible to carry out extensive time course analyses which would have allowed us to define how quickly these proteins were recruited to the intranuclear aggregates. Furthermore, the observed toxicity may also result from partially compromised collective function of several proteins. The impact of sequestration of TRA2β and PA2G4 on their normal cellular functions remains to be determined as an observation of protein co‐localisation within RNA foci is not a definitive indication of their sequestration and loss of function. Indeed, MBNL1 has been shown to co‐localize with CGG inclusions in FXTAS patients,^26^ yet the splicing events coordinated by MBNL1 are not altered in CGG‐expressing cells or in FXTAS patients.^30^ However, we have shown in a premutation mouse model that expresses both CGG‐RNA repeats and FMRpolyG protein that the expression of FUS, PA2G4 and TRA2β are significantly reduced in both granulosa cells and oocytes in all follicle stages, suggesting their deregulation could contribute to disease progression. While these findings do not completely match our observations in HGrC1 cells, it is possible that the older age of these mice (6 months) allows for further disease progression compared with our relatively transient cell line model. These premutation mouse model findings also highlight the importance of studying CGG‐repeat RNA and FMRpolyG in oocytes, though this is complicated by the lack of models available to carry out such investigations.

Together our data support the involvement of an RNA gain‐of‐function hypothesis to the development of FXPOI. While RAN translation of the expanded CGG‐repeat mRNA may also be a contributing factor, these data did not support it as a major cause of granulosa cell death in addition to the effect of the CGG‐repeat RNA only, when investigating only positively transfected cells. In murine models of FXTAS, the expression of FMRpolyG was pathogenic, with these mice exhibiting inclusion formation, motor phenotypes and reduced lifespan, while the sole expression of CGG‐repeat RNA did not induce any of these features^39^; however, no examination of ovarian tissue was carried out. In contrast, Shelly et al (2021) recently studied the fertility phenotype of two premutation mouse models, but in these the expression of coding *Fmr1* was not altered and therefore there was no interference due to decreased FMRP expression.^45^ Only expression of both CGG RNA and FMRpolyG led to a progressive loss of fertility with age^45^ although expression of CGG‐repeat RNA alone was sufficient to impair key ovulatory processes in response to exogenous hormones. CGG RNA foci have not been reported in the ovaries of *Fmr1* premutation mouse models, though it is unclear whether this has truly been explored; our attempts at such experiments were hindered due to the incompatibility between tissue fixation and our CGG FISH protocol. CGG RNA aggregates have also not been reported in women with FXPOI, however FMRpolyG inclusions have been observed in the ovarian stroma of a woman with FXPOI^43^ and the mural granulosa cells of six premutation carriers.^44^ While investigations for CGG RNA aggregates in human FXPOI ovarian tissue have not been undertaken, it is possible that the cellular stress induced by CGG‐repeat RNA and FMRpolyG expression causes atresia of ovarian follicles before RNA aggregates are visible by FISH or immunostaining. Derivation of ovarian somatic cells from FXPOI patient iPSCs may be informative as a model, as these cells will carry the human genetic landscape as well as the disease.^45^

In conclusion, the present data support the involvement of an RNA gain‐of‐function mechanism as a contributing factor to the pathogenesis of FXPOI through the accumulation of large stable nuclear foci formed from expanded CGG‐repeat RNA, which can cause significant granulosa cell death independent of FMRpolyG expression. Furthermore, the identification of proteins that could potentially be deregulated in granulosa cells as a result of interactions with CGG aggregates also supports the involvement of an RNA gain‐of‐function toxicity model, without excluding the contribution of RAN translation‐mediated toxicity.

## AUTHOR CONTRIBUTIONS

Roseanne Rosario, Gracjan Michlewski, Nicholas Charlet‐Berguerand and Richard A. Anderson designed the experiments. Roseanne Rosario, Hazel L. Stewart and Nila Roy Choudhury carried out experiments. Roseanne Rosario wrote the manuscript. All authors contributed to data interpretation, editing the manuscript, and its final approval.

## FUNDING INFORMATION

The authors' work in this field is support by grants from the Wellbeing of Women (PRF005 to RR) and the Medical Research Council (G1100357 to RAA, MR/N022556/1 to the MRC Centre for Reproductive Health) and Biotechnology and Biological Sciences Research Council project grant (BB/T002751/1 to GM). The work was also financed under Dioscuri, a programme initiated by the Max Planck Society, jointly managed with the National Science Centre in Poland, and mutually funded by Polish Ministry of Science and Higher Education and German Federal Ministry of Education and Research (2019/02/H/NZ1/00002 to G.M.). The project was co‐financed by the Polish National Agency for Academic Exchange within Polish Returns Programme as well as National Science Centre (2021/01/1/NZ1/00001 to G.M.).

## DISCLOSURES

RR, HLS, NRC, GM and NCB declare no conflicts of interest. RAA reports grants and personal fees from Roche Diagnostics and Ferring Pharmaceuticals, and personal fees from IBSA, Merck, KaNDy Therapeutics and Sojournix Inc, outside the submitted work.

## Supporting information


Figure S1

Figure S2

Figure S3

Figure S4

Figure S5

Dataset S1

Dataset S2

## Data Availability

The data that support the findings of this study are available in the methods and/or supplementary material of this article.
