# Supplementary material for: Evidence for a fragile X messenger ribonucleoprotein 1 (FMR1) mRNA gain‐of‐function toxicity mechanism contributing to the pathogenesis of fragile X‐associated premature ovarian insufficiency
Source: FASEB J. 2022 Oct 17;36(11):e22612. doi: 10.1096/fj.202200468RR (PMC9828574; doi:10.1096/fj.202200468RR)
Supplement: Supplementary file 1 — Figure S1 [file FSB2-36-0-s002.pdf]

Supplementary figure 1

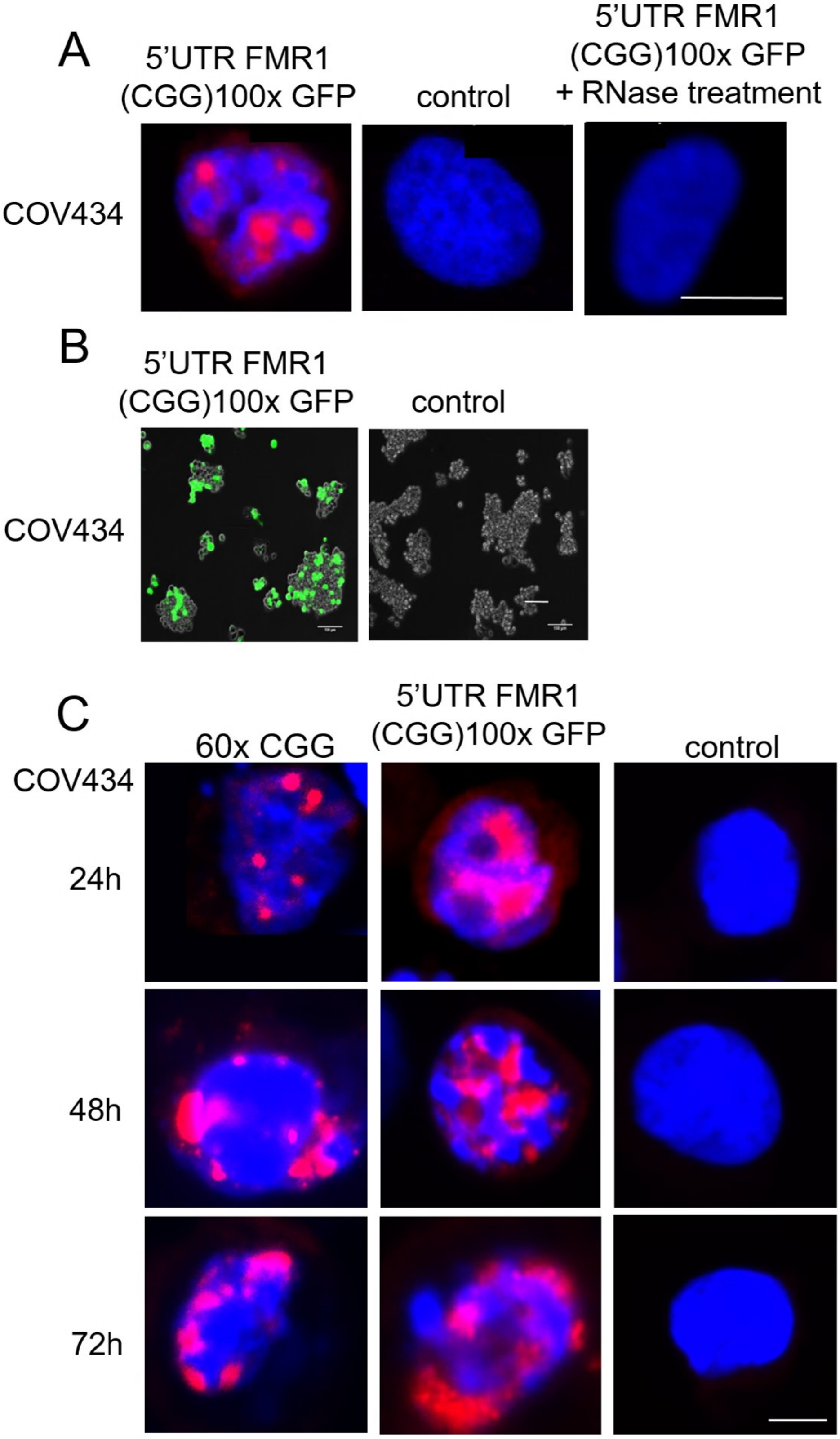


**Supplementary figure 1: Expanded CGG repeats within the *FMR1* 5'UTR form intranuclear RNA aggregates and FMRpolyG protein aggregates in COV434 cells.** COV434 cells were transfected with a plasmid expressing either 100 CGG repeats within the *FMR1* 5'UTR or no CGG repeats (control) and analysed 24h after transfection by RNA FISH using a (CCG)8x-Cy3 DNA probe counterstained with DAPI or fluorescence microscopy for the presence of CGG RNA aggregates (A) or FMRpolyG protein (B), respectively. (C) COV434 cells were transfected with a plasmid expressing either 60x CGG repeats, 100 CGG repeats within the *FMR1* 5'UTR or no CGG repeats (control) and analysed at 24h, 48h and 72h after transfection by RNA FISH. Whilst RNA aggregates formed following expression of 60x CGG repeats increased in size and number over time, RNA aggregates formed following expression of the 5'UTR FMR1 (CGG)100x GFP plasmid were stable in size and number. Scale bars represent 10μM.
