# Supplementary material for: Evidence for a fragile X messenger ribonucleoprotein 1 (FMR1) mRNA gain‐of‐function toxicity mechanism contributing to the pathogenesis of fragile X‐associated premature ovarian insufficiency
Source: FASEB J. 2022 Oct 17;36(11):e22612. doi: 10.1096/fj.202200468RR (PMC9828574; doi:10.1096/fj.202200468RR)
Supplement: Supplementary file 2 — Figure S2 [file FSB2-36-0-s003.pdf]

Supplementary figure 2

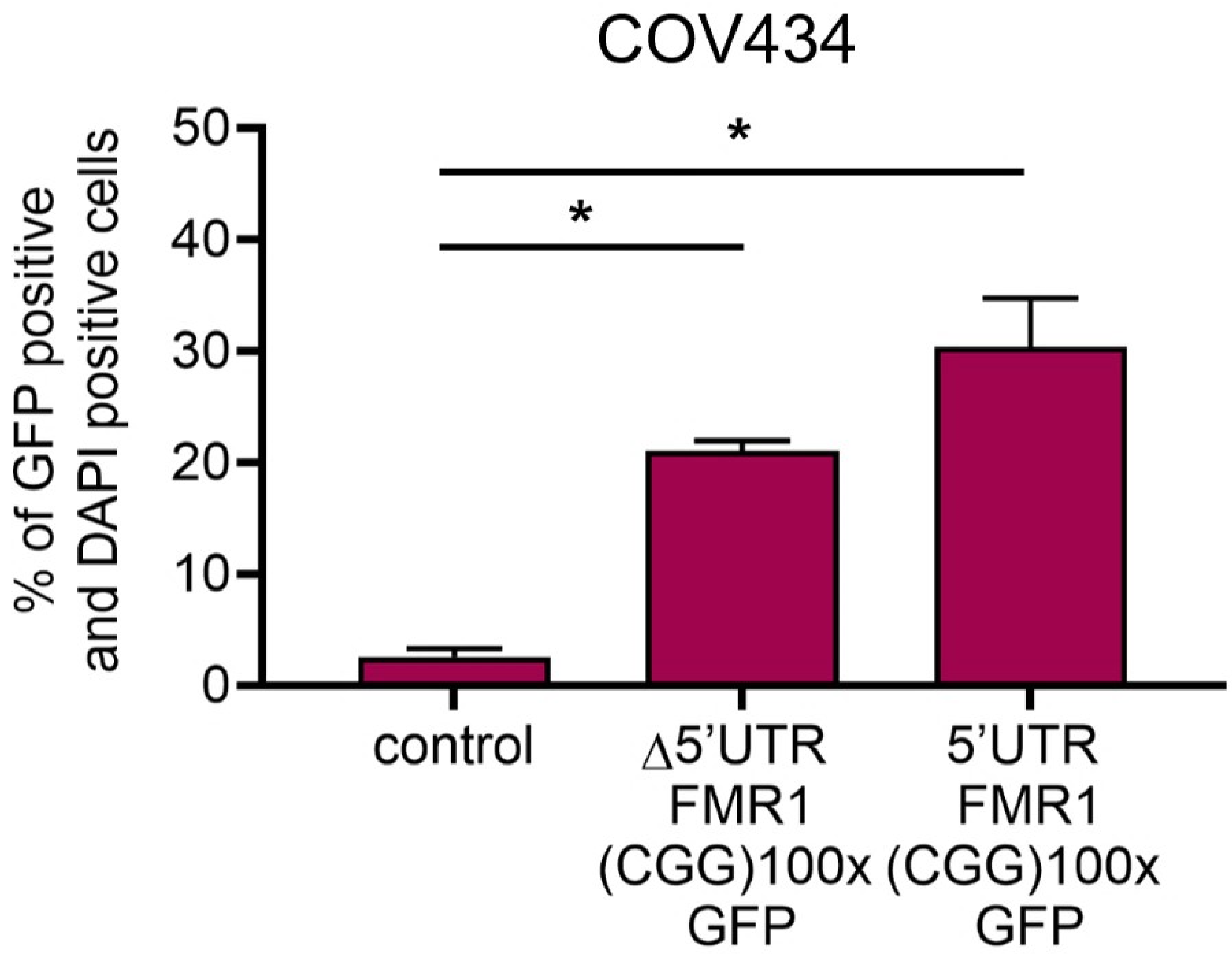


**Supplementary figure 2: COV434 cell viability following expression of CGG-repeat RNA only or CGG-repeat RNA and FMRpolyG.** COV434 cells were transfected with an empty pEGFP plasmid, Δ5'UTR *FMR1* (CGG)100x GFP or 5'UTR *FMR1* (CGG)100x GFP and collected for analysis via flow cytometry at 48h post transfection. Quantification of GFP and DAPI positive COV434 cells. Data are presented as the mean ± SEM of four individual experiments, Mann-Whitney test, *P=0.0286.
