# Supplementary material for: Evidence for a fragile X messenger ribonucleoprotein 1 (FMR1) mRNA gain‐of‐function toxicity mechanism contributing to the pathogenesis of fragile X‐associated premature ovarian insufficiency
Source: FASEB J. 2022 Oct 17;36(11):e22612. doi: 10.1096/fj.202200468RR (PMC9828574; doi:10.1096/fj.202200468RR)
Supplement: Supplementary file 3 — Figure S3 [file FSB2-36-0-s006.pdf]

Supplementary figure 3

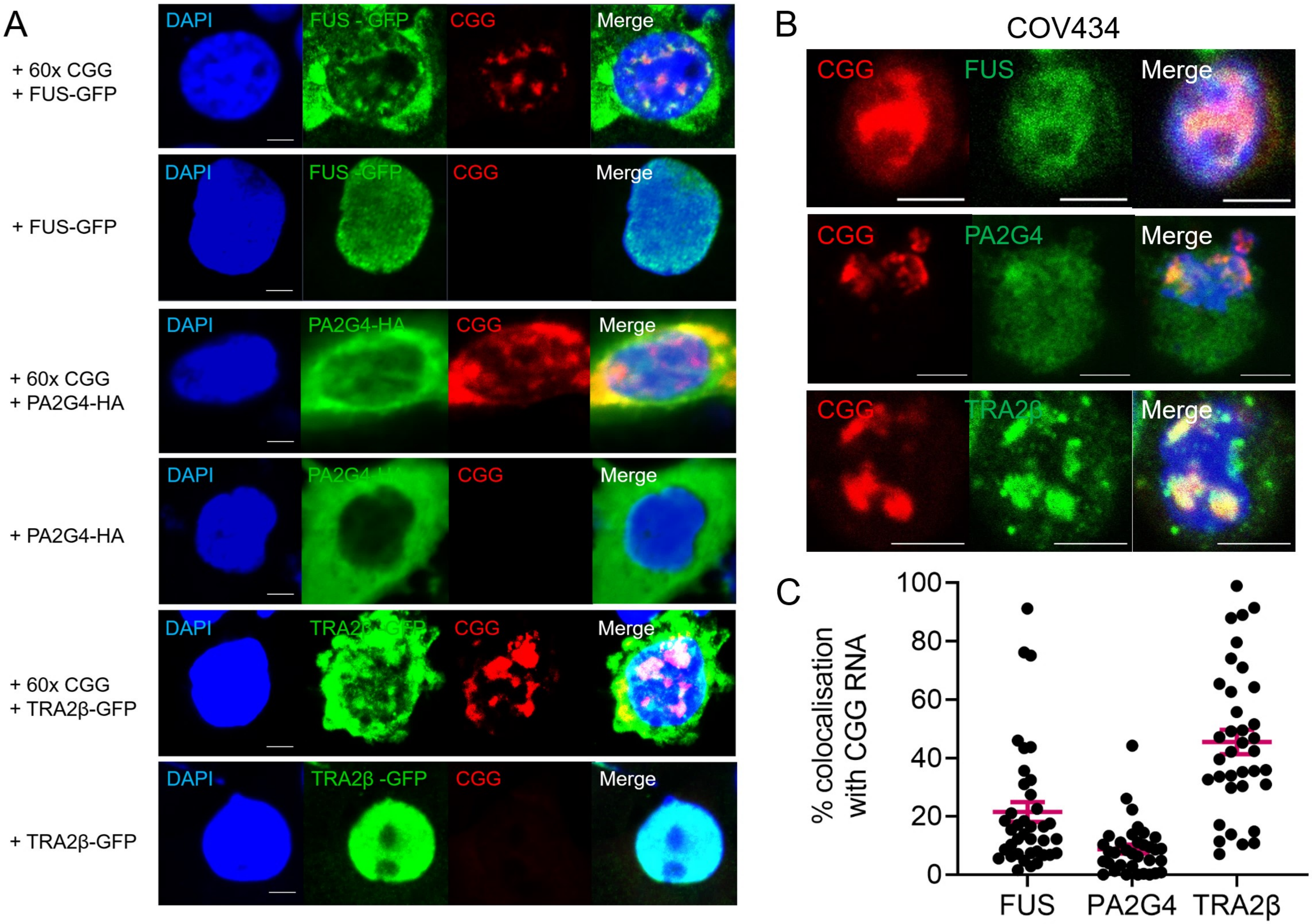

Supplementary figure 3: Co-localisation of exogenously and endogenously expressed FUS, PA2G4 and TRA2β, and 60x CGG-repeat RNA. (A) HGrC1 cells were co-transfected with a plasmid expressing 60x CGG repeats and a plasmid expressing a GFP- or HA-tagged protein of interest, and RNA FISH followed by immunocytochemistry were used 24h after transfection to identify the colocalisation of CGG RNA aggregates and candidate proteins. Scale bars represent 5µM. (B) COV434 were co-transfected with a plasmid expressing 60x CGG repeats and RNA FISH followed by immunocytochemistry were used after 48h to identify the colocalisation of CGG RNA aggregates and candidate proteins. Scale bars represent 10µM. (C) Quantification of colocalisation from 40 individual cells over three separate experiments. Data are presented as the mean ± SEM.
