# Supplementary figures and images for: Evidence for a fragile X messenger ribonucleoprotein 1 (FMR1) mRNA gain‐of‐function toxicity mechanism contributing to the pathogenesis of fragile X‐associated premature ovarian insufficiency
Source: FASEB J. 2022 Oct 17;36(11):e22612. doi: 10.1096/fj.202200468RR (PMC9828574; doi:10.1096/fj.202200468RR)

## Supplementary figure 4

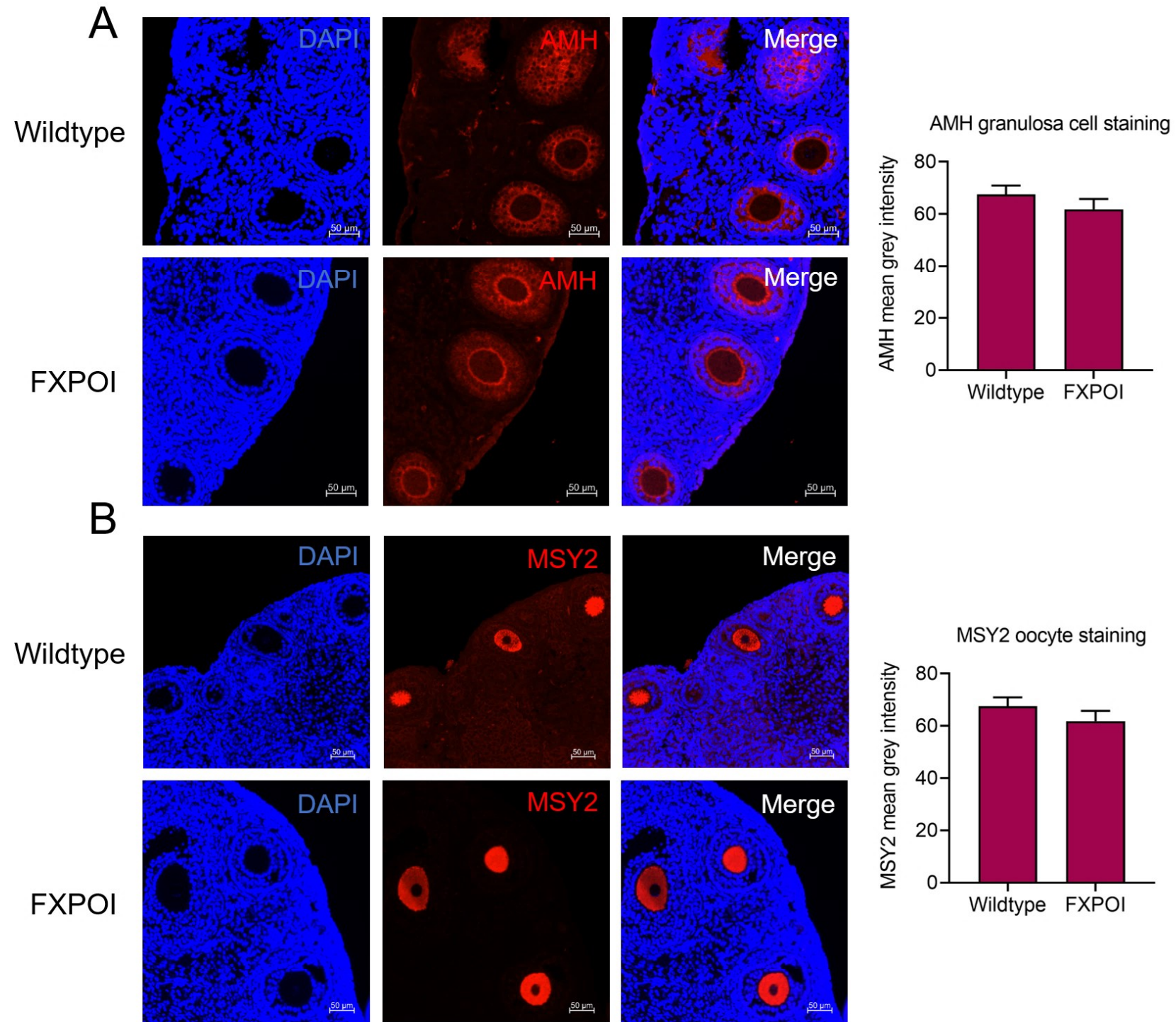

Supplement: Supplementary file 4 — Figure S4 [file FSB2-36-0-s007.pdf]
