# Supplementary material for: Evidence for a fragile X messenger ribonucleoprotein 1 (FMR1) mRNA gain‐of‐function toxicity mechanism contributing to the pathogenesis of fragile X‐associated premature ovarian insufficiency
Source: FASEB J. 2022 Oct 17;36(11):e22612. doi: 10.1096/fj.202200468RR (PMC9828574; doi:10.1096/fj.202200468RR)
Supplement: Supplementary file 5 — Figure S5 [file FSB2-36-0-s004.pdf]

Supplementary figure 5

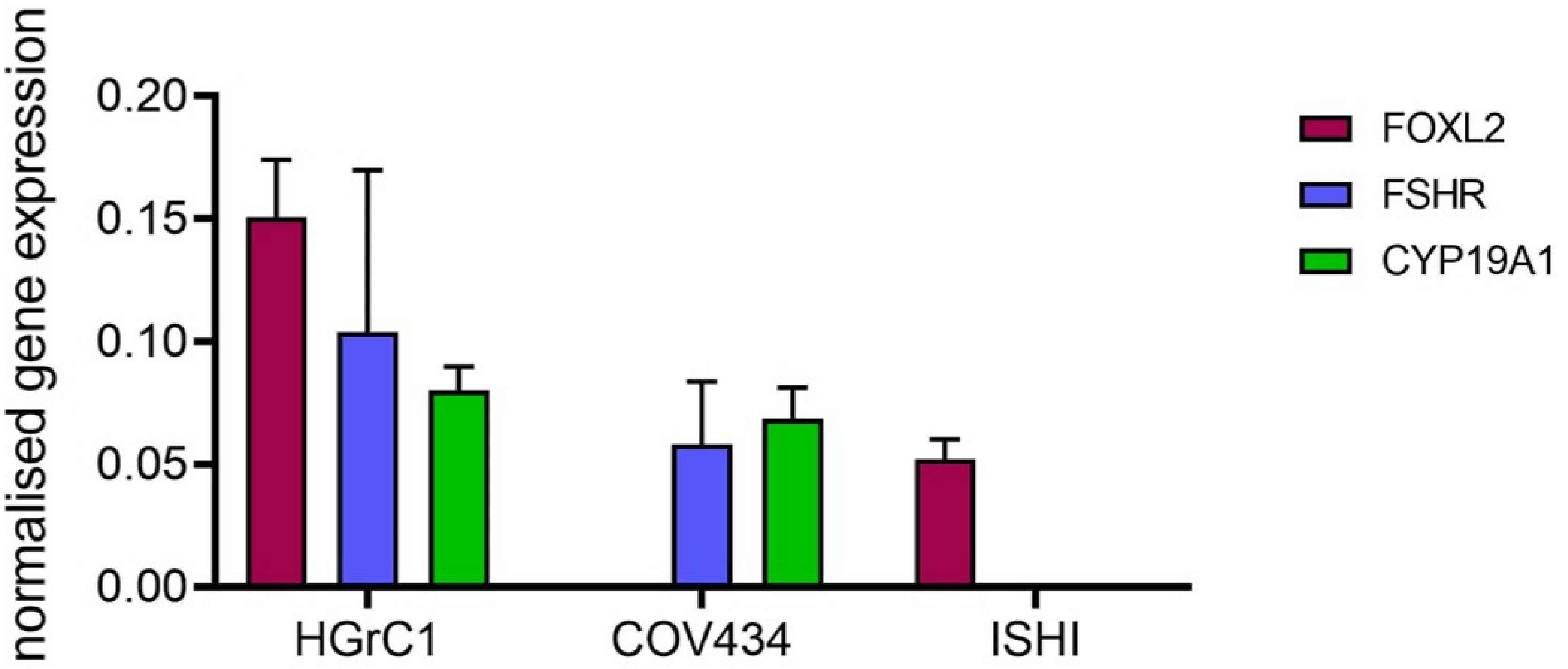

Supplementary figure 5: RT-qPCR characterisation of HGrC1 cells compared to COV434 and Ishikawa (ISHI) cells. HGrC1 cells express *FOXL2*, *FSHR* and *CYP19A1*. COV434 cells also expressed *FSHR* and *CYP19A1*, but lacked *FOXL2* expression, a finding which has been reported by ourselves previously (65). Ishikawa cells were chosen for comparison as they are an endometrial carcinoma cell line, and thus represent another female reproductive tissue cell line. *FOXL2* expression has been observed in human endometrial cells, where it is thought to regulate endometrial receptivity (66-68). Error bars indicate ± standard deviation of technical replicates.
